# Paraplegia secondary to disseminated mucormycosis: case report and literature review

**DOI:** 10.1186/s12879-022-07373-8

**Published:** 2022-04-25

**Authors:** Xiangjun Shi, Lei Qi, Boran Du, Xingchen Yao, Xinru Du

**Affiliations:** 1 Department of Hematology, Beijing Chao-Yang Hospital, Capital Medical University, Beijing, China; 2 Department of Orthopaedics, Beijing Chao-Yang Hospital, Capital Medical University, Beijing, China; 3 Department of Pharmacy, Beijing Obstetrics and Gynecology Hospital, Capital Medical University, Beijing, China; 4 School of Medicine, Tsinghua University, Beijing, China

**Keywords:** Case report, Disseminated mucormycosis, Spine involved, Paraplegia, Surgical treatment

## Abstract

**Background:**

We report a case of spine infection with mucormycosis that manifested signs of paraplegia in a patient suffering from disseminated mucormycosis. Timely and effective surgery was performed. A review of the literature is included.

**Case presentation:**

A patient with diabetic ketoacidosis complained of back pain and fatigue for one month, and his right lower extremity activity had been limited for 10 days. T4–T6 vertebral and paravertebral soft tissue-involved infections were identified by MRI, which were derived from right lung pneumonia. He underwent abscess debridement, spinal canal decompression, pedicle screw fixation and amphotericin B liposome injection. Histopathological examination revealed broad aseptate hyphae suggestive of invasive mucormycosis. There was improvement in neurological function after surgical and medical treatment. Three months after the surgery, the patient died of uncontrollable massive bleeding of the urinary system. Mucormycosis is characterized by rapid development and a high mortality rate. This case shows the significance of a multidisciplinary team in the diagnosis and treatment of patients with mucormycosis. In addition, orthopedic surgeons should design appropriate surgery plans for spine-involved mucormycosis patients.

**Conclusion:**

This case present a patient with paraplegia caused by the spread of pulmonary mucormycosis to the vertebral and paravertebral soft tissue of levels T4–T6. After medical treatment, surgical debridement and internal fixation, the patient recovered well but later patient died of possible disease dissemination to the renal or urinary tract which resulted in massive haemorrhage.

## Background

Mucormycosis, previously known as zygomycosis or phycomycosis, is a rare and opportunistic fungal infection characterized by rapid development and a high mortality rate [1]. The causative agents of mucormycosis often belong to the genus *Rhizopus*, *Lichtheimia, Mucor* and *Cunninghamella* [2, 3]. Mucormycosis is most common in patients with seriously compromised immune systems, poorly controlled diabetes and ketoacidosis, hematologic malignancies, immunosuppressive disorders, solid organ or bone marrow transplantation hence forth [4]. The portals of entry of the organisms include inhalation, ingestion, and skin penetration caused by accidental wounds, extensive bums, central venous catheters, or surgery. Uncontrolled diabetes and diabetic ketoacidosis were susceptible to Mucorales. Patients with diabetic acidosis who inhale mucor spores can easily develop pulmonary mucormycosis. Nevertheless, no obvious invasion site can be found in some cases [5].

According to the region of infection, mucormycosis is divided into six types: rhinocerebral (most common), pulmonary, cutaneous, gastrointestinal, disseminated and other rare forms, such as osteomyelitis, peritonitis, endocarditis and renal infection [6]. Spinal or paraspinal tissue-involved mucormycosis is especially rare. To date, only 10 cases have been reported, of which only 3 cases were treated with surgical debridement. Here, we review the patient's data and systematically review the relevant literature.

## Case presentation

**Fig. 1:**
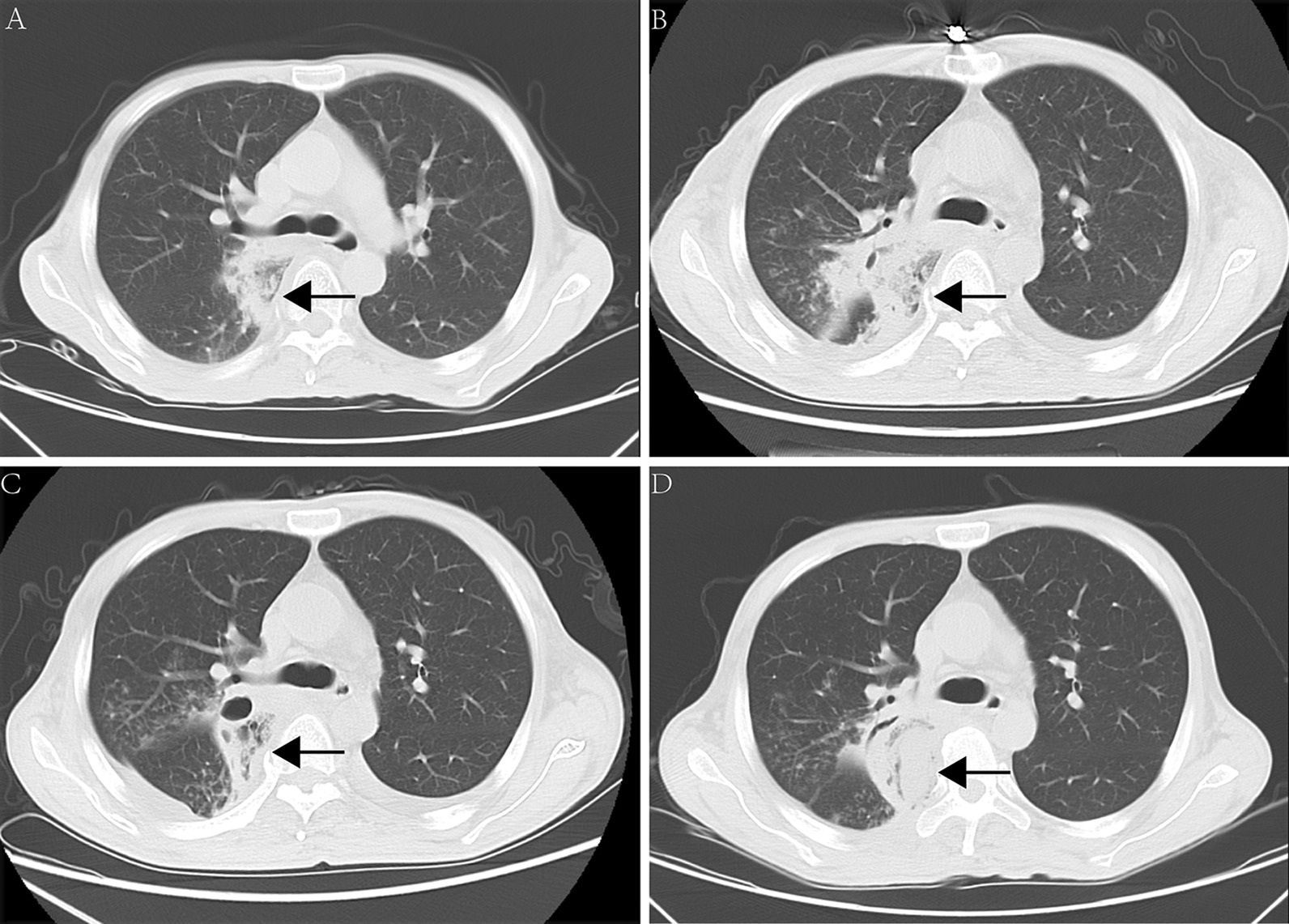
**A** High-density image and area without lung markings next to the hilus of the right lung. **B** The area of high-density shadow was obviously enlarged, and pleural effusion appeared on both sides. **C** The pneumonia had been absorbed partly as a central area without any obvious changes, but the pleural effusion had increased in both lungs. **D** The paraspinal lesion continued to grow, the residual lung tissue disappeared completely, the bilateral pleural effusion shrank significantly, but the bone did not change obviously

**Fig. 2:**
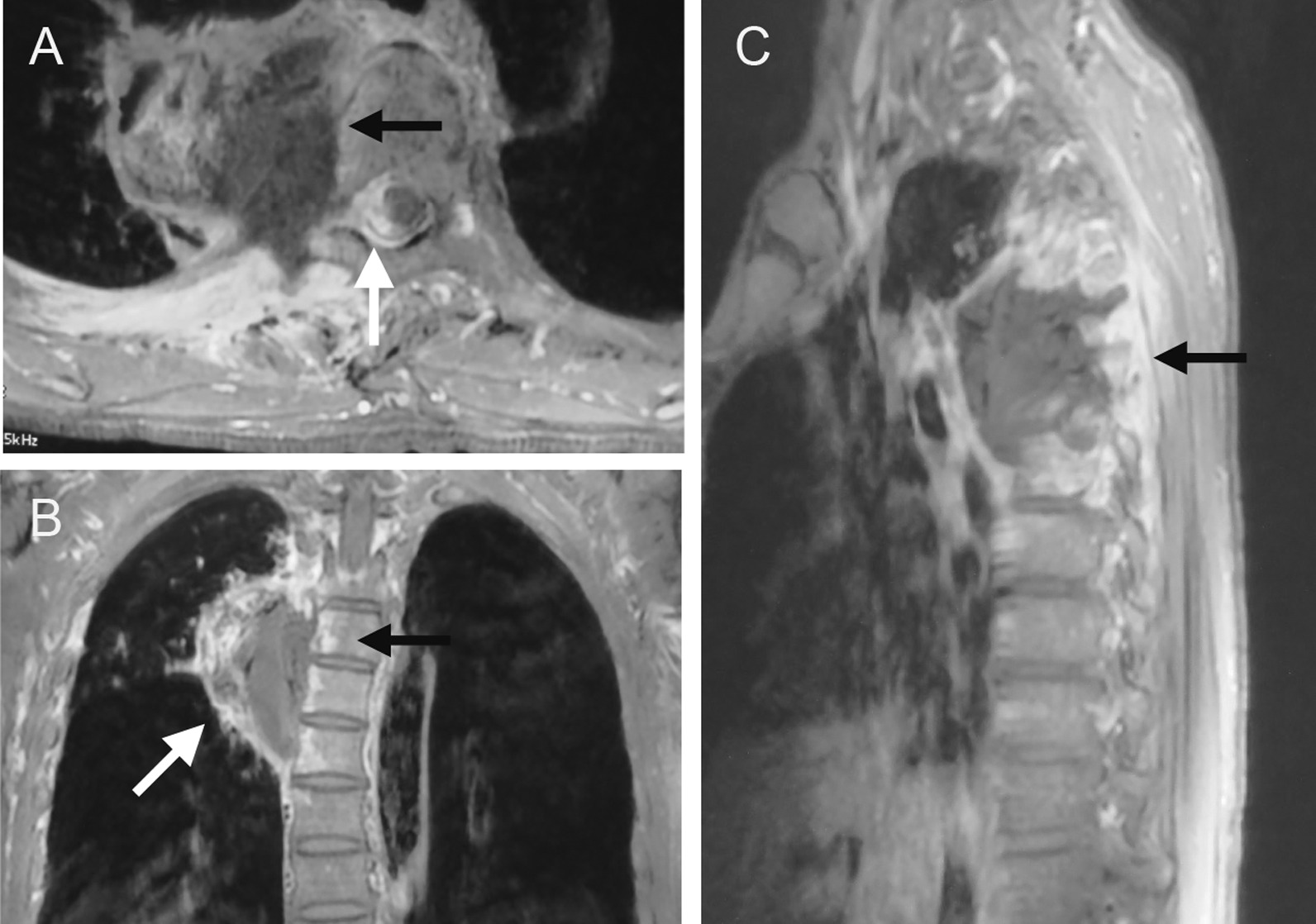
**A** On the right side of the spine, the lesion showed short hypointensity on T2 and was surrounded by high-signal T2 changes (black arrow). The spinal cord was compressed to the left by the abscess (arrow). **B** and **C** The abscess (white arrow) next to the T4–T6 segments surrounded by hyperintense tissue. The vertebrae were hyperintense (black arrow)

A 48-year-old Asian male who had a history of diabetes was diagnosed with diabetic ketoacidosis and admitted to another hospital. He complained of upper back pain seven days before admission, accompanied by fatigue, nausea and a dripping feeling when urinating for four days. The blood glucose was 19.8 mmol/L (normal: 3.9–6.2 mmol/L), and the pH was 6.823 (7.35–7.45). The body temperature was 36 °C. Patient presented with altered mental status. The first chest CT (Fig. 1A) suggested inflammation of the right lung. Procalcitonin = 5.22 ng/mL (normal <  0.05 ng/mL), HR-CRP = 223.05 mg/L (normal: 0–3.0 mg/L), WBC = 8.48 × 10^9^/L (normal: 4–10 × 10^9^/L), and NEU = 83.84% (normal: 50–70%). After application of moxifloxacin, rehydration, insulin and the correction of his ketosis and electrolyte disorder for 2 days, his body temperature was 38.5 °C. Cefoperazone (2.0 g, q12h) was given for 10 days, during which his body temperature was between 36.4 and 37.6 °C, PCT was 0.104 ng/mL, HR-CRP was 57.03 mg/L, WBC was 7.38 × 10^9^/L (normal: 4–10 × 10^9^/L), and NEU was 83.21%. The second CT examination (Fig. 1B) revealed that the infection was further aggravated, the range of infection was enlarged, and effusion appeared in the bilateral pleural cavity. Oral moxifloxacin (0.4 g, qd) combined with cefoperazone/sulbactam (2.0 g, q12h) was applied. On the 18th day of admission, the patient complained of weakness of the right lower extremity and positive Babinski's sign on the right side, and the muscle strength of the right lower extremity was decreased to grade 4/5. The third chest CT (Fig. 1C) indicated that the lesion was larger than ever and that the bilateral pleural effusion had increased. Three weeks after admission, the cefoperazone/sulbactam was discontinued, and azithromycin (0.5 g, qd) was administered. The patient’s symptoms rapidly worsened, and the muscle strength of the right lower extremity decreased to grade 1/5. The fourth chest CT (Fig. 1D) showed that the extent of the paraspinal abscess lesions was larger and that the pleural effusion had increased. MRI (Fig. 2A–C) suggested abnormal signals in the T3–T6 vertebrae, a compressed thoracic spinal cord and the formation of an abscess in the right lung. Laboratory examination on the 35th day of admission showed that the white blood cell count was 10.11 × 10^9^/L, total protein was 64.9 g/L (normal: 60–80 g/L), albumin was 36.6 g/L (normal: 40–55 g/L), serum potassium was 3.43 mmol/L L (normal: 3.5–5.5 mmol/L), ferritin was normal, and procalcitonin was 0.062 ng/mL. Microbiological and pathological examinations of the bronchoalveolar lavage fluid were negative. His blood glucose was13.8 mmol/L.

**Fig. 3:**
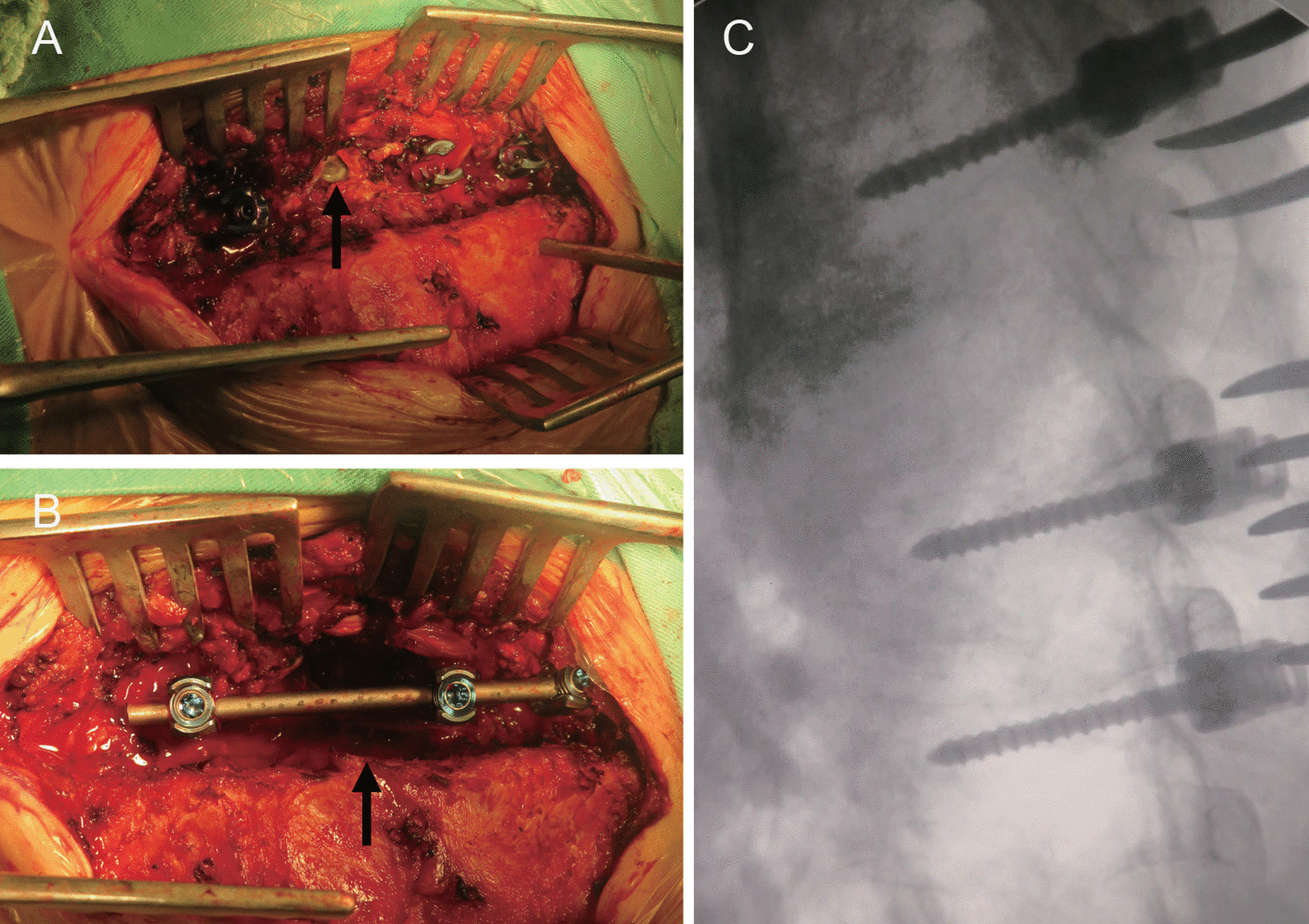
**A** Purulent fluid can be seen in the area indicated by the black arrow. **B** The right laminectomy of T4. The fester and the necrotic tissue in the spinal canal were removed (black arrow). **C** Pedicle screws were inserted on the right side of T3, T5, and T6, and a rod was placed

At this time, the patient was admitted to our hospital. The physical examination indicated tenderness in the T4–T5 spinous process, the muscle strength of the right lower limb was grade 1/5, that of the left lower limb was grade 3, hyperreflexia of the tendon was obvious, and Babinski's sign on the right side was positive. Tazocin (4.5 g, q8h) was given empirically, and posterior thoracic abscess debridement, spinal canal decompression and pedicle screw fixation were performed the next day (Fig. 3A–C). The abscess was wrapped around the right side of the vertebral body of T4 and T5 and communicated with the epidural space through the intervertebral foramen. The paraspinal muscles showed edema, and 20 mL of purulent fluid was removed. The right laminectomy in T4 and spinal canal decompression were performed because the infection was located on the right side of the thoracic vertebra and the contralateral joint was stable. A pedicle screw internal fixation system was installed only on the right side of the T3, T5, and T6 segments. Two tubes were put in for drainage at the upper and lower ends of the incision, respectively.

**Fig. 4:**
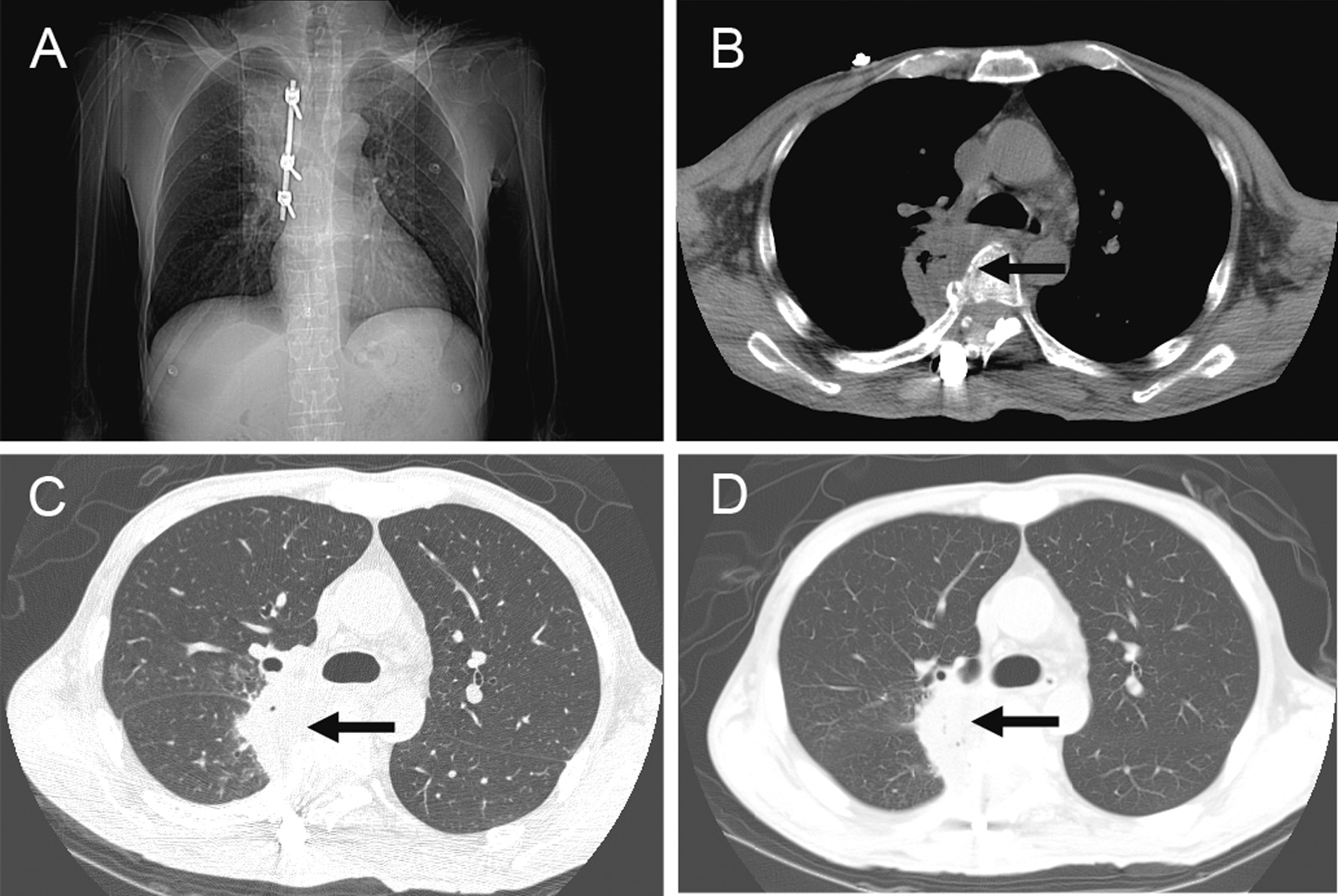
The right lung abscess still existed, but the pneumonia was alleviated (black arrow). **A** Chest CT images were reviewed 5 days after the operation. **B** Chest CT images were reviewed 5 days after the operation. The abscess cavity decreased and pleural effusion disappeared. **C** and **D** Reexamination at 16 and 21 days after surgery showed no significant changes in lung inflammation

Cefoperazone sulbactam sodium 4.5 g/days was infused intravenously after the operation. Five days later, chest CT showed a large shadow on the upper lobe and paraspinal side of the right lung with a size of approximately 4.9 × 4.2 cm. No significant changes were observed among the CT scans on the 5th (Fig. 4A and B), 16th (Fig. 4C) and 21st days after operation (Fig. 4D).

**Fig. 5:**
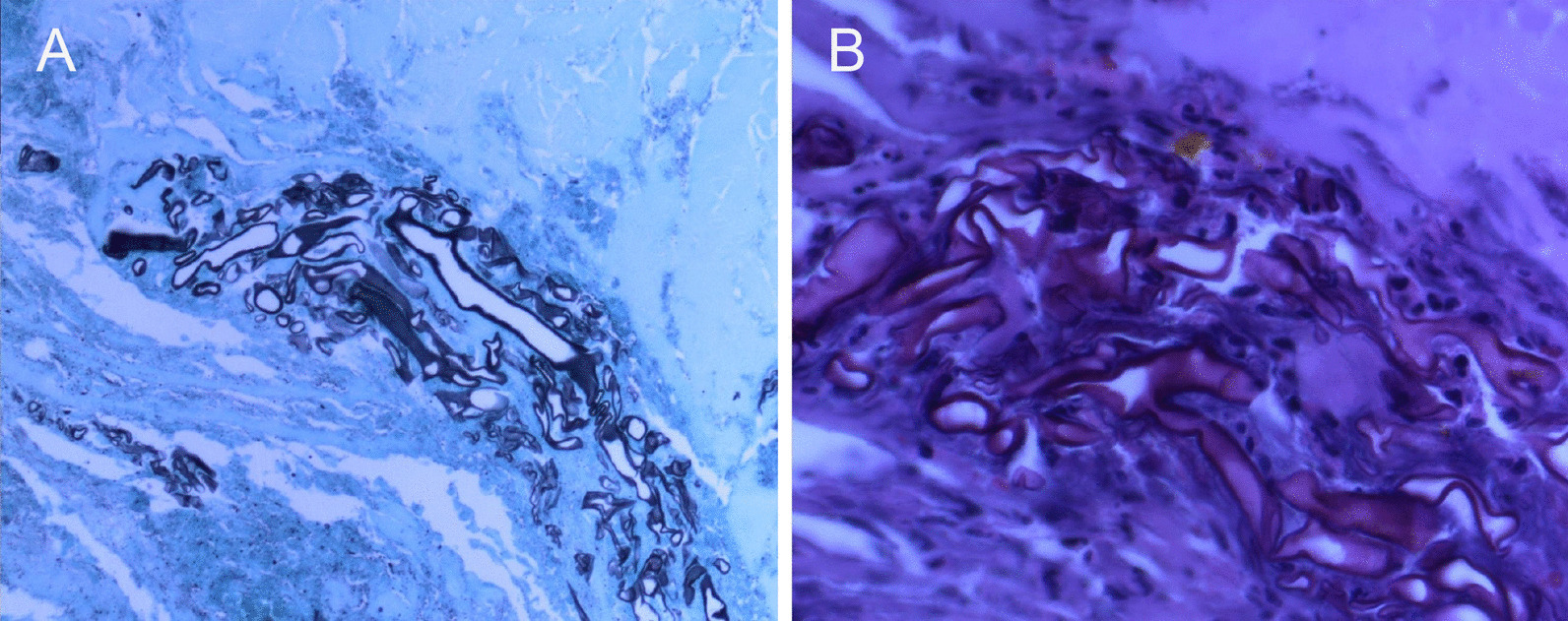
Typical mucoraceous hypha with atactic and cross branches arising from the parent hypha at right angles. **A** PAS, × 200; **B** HE, × 400. Images were obtained using a Nikon Eclipse 200 fluorescence microscope (NIS Elements 2.x software)

On the 23rd day after operation, the pathology results (Fig. 5A and B) indicated that mucormycosis. Given this pathology, amphotericin B liposomes 50 mg/days and posaconazole 600 mg/days were applied, and the temperature and blood glucose of the patient were generally normal.

The patient was given amphotericin B liposomes 100 mg/days and posaconazole 600 mg/days. Considering the high mortality rate of mucormycosis. Ten days later, the amphotericin B liposomes were adjusted to 80 mg/days. Two months after the operation, the patient was discharged from the hospital, and the incision was healing well, with grade 3/5 muscle strength of the right lower limb and grade 4/5 muscle strength of the left lower limb. The Frankle grade recovered to D, and the patient could walk with crutches.

Three months after the operation, we were informed that the patient died of uncontrollable bleeding of the urinary system. The cause of death was possibly due to the dissemination of the disease.

## Discussion and conclusion

Mucor spores are ubiquitous in the natural environment. If healthy people inhale spores, macrophages and neutrophils phagocytose the oxidized spores to prevent fungal invasion. However, these cells cannot inhibit spore germination and kill hyphae effectively in immunocompromised patients (those with neutropenia, diabetic ketoacidosis, immunosuppressive therapy and so on). Disorders of these cells are key factors in vascular invasion [7]. Our patient suffered from diabetes, ketoacidosis and pulmonary mucormycosis, in line with the literature. Nevertheless, mucormycosis is seldom seen in vertebral or paravertebral soft tissue. To the best of our knowledge, osteomyelitis caused by mucor infection is uncommon but has been described in the tibia, femur, humerus, scapula, metacarpal, phalanx, sternum, cuboid, calcaneus, tibia, internal fixation and repaired anterior cruciate ligament [8]. Mucormycosis spondylitis is very rare in clinical practice. To date, only 10 literatures of mucormycosis osteomyelitis have been reported in the English-language literature, according to a search of PubMed, Embase. The data of all these cases are summarized in Table 1. The average age of patients with spinal mucormycosis involvement is 50.2 years. Males account for 63.6%, and the mortality rate of patients with spinal involvement is 63.6%, slightly higher than the 54% reported in the literature for mucormycosis without spinal involvement [9]. Four patients have suffered from diabetes (2 with ketoacidosis), 2 malignant tumors, 1 MDS (myelodysplastic syndrome), 2 renal insufficiency, 1 hypohepatia, and only 1 without underlying disease. In our case, thoracic spine involvement, vertebral osteomyelitis, spinal cord compression and paraspinal soft tissue abscess were evidenced. Approximately 2 months after the symptoms the disease was diagnosed as mucormycosis (35 days in first hospital and 23 days in second) which also showed that delay in diagnosis and initiation of appropriate therapy with antifungals was the major reason for dissemination of the disease. Delays to diagnosis and treatment can be a possible cause of disease transmission and death. In conclusion, the thoracic vertebrae are the most easily affected sites in mucormycosis spondylitis, while cervical or sacral involvement is unusual. Special attention should be given when the suspected diagnosis is mucormycosis spondylitis and timely antifungals treatment should be given.

**Table 1:** Summary of all reported cases of spine-involved mucormycosis

Number	Year	Author	Age/sex	Presenting symptoms	Spinal involvement Position	Underlying conditions	Treatment	Primary lesion	Outcome
1	2018	Present	48/M	Paralysis, fever	T3–T6	Diabetic ketoacidosis	Surgical debridement, posaconazole, amphotericin B liposomes, spinal canal decompression, pedicle screw fixation	Lung	Dead
2	2017	Shah et al. [20]	54/M	Mechanical low back pain, right lower limb radiation	L3–L4	Cryptogenic liver cirrhosis,	Liposomal Amphotericin B	None	Dead
3	2016	Wang et al. [16]	20/F	Lower extremity numbness and weakness fever, dysuria	T3–T6	Right lung pneumonectomy	Amphotericin B	Chest wall	Live
4	2015	Navanukroh et al. [13]	42/F	Dry cough, left buttock and left lower limb sharp shooting pain, fever	S1	Kidney transplantation	Decompressive laminectomy of two segments, liposomal amphotericin B	Lung	Live
5	2015	Hadgaonkar et al. [8]	64/M	Low back pain, fever, weight loss	L4, L5	Diabetes mellitus, hypertension, chronic kidney disease	Amphotericin B	None	Dead
6	2010	Giuliani et al. [5]	54/F	Paraparesis	T10–T12	Acute septic panniculitis, decompensated Diabetes mellitus, obliterant arteriopathy	Acyclovir, Liposomal Amphotericin B	None	Live
7	2006	Chen et al. [12]	57/F	Low back pain, fever, weakness and numbness of the lower extremities	L4, L5	None	Surgical debridement, amphotericin-B	None	Live
8	2000	Machida et al. [11]	58/M	Fever, paraplegia	T12, L1	Acute myelocytic leukemia	Broad-spectrum antibiotics, amphotericin-B Local irradiation	Lung	Dead
9	1996	Pohle et al. [19]	43/M	Lower extremity weakness, fever, weight loss	T3, T4	Diabetic ketoacidosis, pancreatitis	Amphotericin B	Lung	Dead
10	1988	Rozich et al. [14]	52/M	Back pain, lower extremity weakness, fever	L1–L2	MDS, splenectomy	Amphotericin B, Surgical debridement	Lung	Dead
11	1979	Buruma et al. [10]	60/M	Neck pain, arm weakness	C3, C4	Carcinoma of the hypopharynx	None	None	Dead

Most mucormycosis spondylitis cases are not treated with surgery. Buruma et al. [10] reported the first mucormycosis spondylitis patient, who had a history of laryngectomy and radiotherapy and became paralyzed as a result of cervical mucormycosis. The patient died of pulmonary embolism and cervical spondylitis of mucormycosis, which was confirmed by autopsy and pathological examination. Hadgaonkar et al. [8] reported isolated mucormycosis spondylitis in a patient with a history of diabetes, hypertension, chronic nephropathy and dialysis. The patient's MRI showed that only the L4–L5 vertebrae had an abnormal signal intensity, and no other lesions existed. Mucor infection was confirmed by lumbar biopsy. The patient died of septicemia and multiple-organ failure. Machida et al [11] reported a patient with myelodysplastic syndrome associated with subacute myelopathy who received local radiotherapy, which was ineffective. Autopsy confirmed that fungal exudates blocked the anterior vertebral artery and resulted in spinal cord infarction. Giuliani et al. [5] reported a case of an infection in the thigh and hip skin that disseminated to the T10–T12 vertebrae. CT showed no bony destruction, but MRI showed osteomyelitis. Paraplegia was caused by spinal cord infarction due to obstruction of the arteriae spinalis anterior. After debridement of the skin and subcutaneous infection, the patient was discharged from the hospital after 3 months, without any recovery of the paraplegic symptoms.

Only 3 patients received surgical debridement of infected vertebral and paravertebral soft tissue. Chen et al. [12] reported a case of lumbar vertebrae mucormycosis after disc puncture. The patient underwent repeated posterior debridement. The patient eventually recovered with oral medication for another 8 weeks. Both Navanukroh et al. [13] and Rozich et al. [14] reported similar cases of disseminated primary lung mucormycosis that spread into the lumbar vertebrae. Both patients underwent posterior necrotic tissue debridement, laminectomy and decompression of the spinal canal. One patient died, and the other patient survived. The case we present is the first case in which thoracic vertebrae were involved in mucormycosis. He underwent posterior spinal surgery. The patient developed severe pulmonary infection and rapid progression of paraplegic symptoms, so we performed debridement and decompression. After timely operation and intravenous injection of amphotericin B, which was effective against the Mucor, the patient was generally in good condition, and his paraplegia symptoms partly recovered. The reason for the incomplete recovery might be that Mucor invades and blocks blood vessels, which results in ischemic and irreversible damage to the spinal cord. If such a case does not permit surgery as early as possible, irreparable sequelae will result [5]. Regrettably, the patient died of bleeding of the urinary system due to aggravation of residuary lung lesions.

Mucormycosis spondylitis and paraspinal abscess have different clinical manifestations. As described in Table, the general symptoms of mucormycosis spondylitis include malaise, weight loss, fever, local symptoms of pain (most common), nerve damage (radiculopathy, myelopathy and cauda equina syndrome), paralysis, etc. [15]. It is difficult to distinguish mucormycosis spondylitis from other spondylitis through imaging. Mucormycosis spondylitis cannot be diagnosed directly by clinical manifestations, laboratory examination and imaging. The positive rate of tissue culture is not high, so a definite diagnosis requires biopsy and confirmation of the special morphology of the hyphae [16]. In this case, the appearance of paraplegic symptoms was caused by direct invasion of the primary pulmonary mucormycosis. Etiological tests were negative, empirical use of antibiotics was inefficient, and the specific type of spinal infection could not be clarified by imaging. The correct diagnosis depends on the observation of specific hyphae in debridement tissue on pathological examination.

Therapeutic measures to treat mucormycosis include early diagnosis, systemic antifungal therapy, basic disease control and aggressive surgical debridement [17]. To date, the survival rates have increased since amphotericin B has been introduced to treat mucormycosis, but they are still low. In the study of Roden et al. [9], surgical debridement was an independent risk factor for survival rates in patients with mucormycosis. Mucor shows vascular invasiveness and blocks the vascular lumen with mycelium and exudates, which makes antifungal drugs ineffective [3, 18]. Therefore, surgical debridement is more important for curing the disease. For patients with spinal cord compression, surgical debridement not only saves patients' lives but also benefits their functional recovery and the improvement of their quality of life as soon as possible.

Internal fixation has a positive effect on maintaining spinal stability and facilitating the clearing of infections in spinal infectious diseases [15]. To our knowledge, this is the first patient who underwent unilateral laminectomy, decompression of the spine and pedicle screw implantation. An internal fixation device was applied for mucormycosis spondylitis, and the patient recovered well. Unfortunately, due to the sudden death of the patient, long-term follow-up on this surgical method was impossible. There is no consensus about whether to use an internal fixation device and what kind of operation should be carried out for this disease among experts in various countries, so it is necessary to study more cases and large samples of randomized patients.

Spinal or paraspinal tissue-involved mucormycosis is seldom seen in the clinic and has a high mortality rate. Doctors must remain alert and aware of scarce spinal infectious diseases and do comprehensive clinical evaluations and pathological biopsies to diagnose them early. Multidisciplinary team diagnosis and treatment is particularly valuable for such diseases. It mainly involves surgery, internal medicine to adjust blood glucose, infection department to anti-infection treatment. Orthopedic surgeons should design appropriate and individualized surgery plans for spine-involved mucormycosis patients.

## Data Availability

The datasets used and analyzed during the current study are available from the corresponding author on reasonable request.

## Abbreviations

MRI: Magnetic resonancediffusion tension imaging
QD: Quaque die
CT: Computerized tomography imaging
HR-CRP: Hypersensitive C-reactive protein
WBC: White blood cells
NEU: Neutrophile granulocyte
PCT: Procalcitonin
MDS: Myelodysplastic syndrome
